# A Plasma Proteomic Signature of Skeletal Muscle Mitochondrial Function

**DOI:** 10.3390/ijms21249540

**Published:** 2020-12-15

**Authors:** Marta Zampino, Toshiko Tanaka, Ceereena Ubaida-Mohien, Giovanna Fantoni, Julián Candia, Richard D. Semba, Luigi Ferrucci

**Affiliations:** 1 National Institute on Aging, National Institutes of Health, Baltimore, MD 21224, USA; marta.zampino@nih.gov (M.Z.); tanakato@mail.nih.gov (T.T.); ceereena.ubaida-mohien@nih.gov (C.U.-M.); 2 Clinical Research Core, National Institute on Aging, National Institutes of Health, Baltimore, MD 21224, USA; giovanna.fantoni@nih.gov; 3 Laboratory of Human Carcinogenesis, Center for Cancer Research, National Cancer Institute, National Institutes of Health, Bethesda, MD 20892, USA; julian.candia@nih.gov; 4 Wilmer Eye Institute, Johns Hopkins University School of Medicine, Baltimore, MD 21287, USA; rdsemba@jhmi.edu

**Keywords:** oxidative capacity, mitochondria, skeletal muscle, inflammation, plasma, proteomics, aptamers, SOMAscan, phosphorous magnetic resonance spectroscopy

## Abstract

Although mitochondrial dysfunction has been implicated in aging, physical function decline, and several age-related diseases, an accessible and affordable measure of mitochondrial health is still lacking. In this study we identified the proteomic signature of muscular mitochondrial oxidative capacity in plasma. In 165 adults, we analyzed the association between concentrations of plasma proteins, measured using the SOMAscan assay, and skeletal muscle maximal oxidative phosphorylation capacity assessed as post-exercise phosphocreatine recovery time constant (τ_PCr_) by phosphorous magnetic resonance spectroscopy. Out of 1301 proteins analyzed, we identified 87 proteins significantly associated with τ_PCr_, adjusting for age, sex, and phosphocreatine depletion. Sixty proteins were positively correlated with better oxidative capacity, while 27 proteins were correlated with poorer capacity. Specific clusters of plasma proteins were enriched in the following pathways: homeostasis of energy metabolism, proteostasis, response to oxidative stress, and inflammation. The generalizability of these findings would benefit from replication in an independent cohort and in longitudinal analyses.

## 1. Introduction

Mitochondrial oxidative phosphorylation is the major source of energy production for all cellular functions [1]. Accordingly, impaired mitochondrial function, one of the hypothetical mechanisms that drives the aging process [2], has been associated with the development of phenotypical and functional manifestations of aging and with age-related diseases [3,4,5]. Mitochondrial oxidative capacity measured in vivo in skeletal muscle declines with aging and is associated with lower walking speed, muscle strength, and physical activity independent of age [6,7,8], as well as with chronic inflammation [9]. Consistent with the decline of oxidative capacity with aging, discovery proteomic studies in skeletal muscle from healthy individuals over a wide age-range have shown a substantial decline of mitochondrial proteins with aging, including proteins of electron transport chain complexes (ETC), enzymes of the Krebs cycle as well as structural proteins [10]. In a recent study, aimed to define the proteomic signature of mitochondrial oxidative capacity in skeletal muscle, we identified muscle proteins that were differentially represented in individuals with higher and lower oxidative capacity, measured by phosphorus magnetic resonance spectroscopy (^31^P-MRS) [11]. As expected, proteins overrepresented in muscle with higher oxidative capacity were enriched for pathways connected with mitochondrial metabolism and translation within mitochondria. Unexpectedly, we also found highly significant enrichment for mRNA processing/alternative splicing pathways, although this finding remains unexplained [11].

Although the identification of specific skeletal muscle proteins that are associated with a direct measure of mitochondrial function is important to gain insight into mechanisms of mitochondrial decline, it has limited clinical use because it is invasive and requires muscle biopsy specimens. In this study, we hypothesized that reduced oxidative capacity in skeletal muscle may be reflected by characteristic changes in circulating proteins and, therefore, we searched for a proteomic signature of muscular mitochondrial oxidative capacity in plasma.

## 2. Results

**Figure 1:**
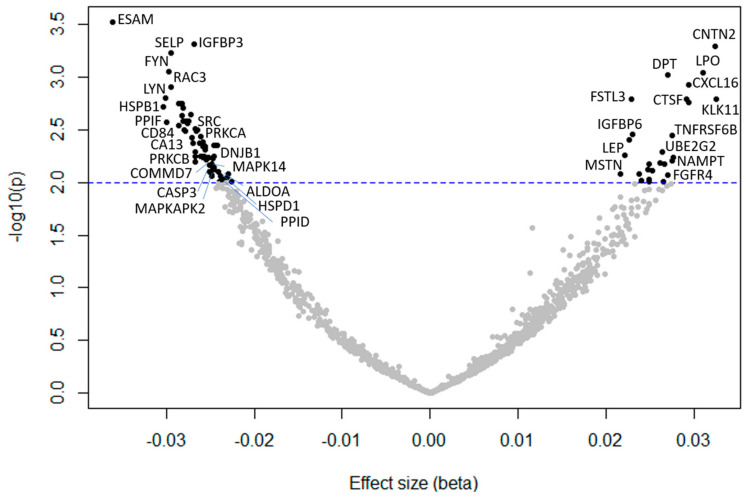
Volcano plot of the association between protein concentrations (gene ID in the plot) and mitochondrial oxidative capacity (τ_PCr_), adjusted for age, sex, and phosphocreatine depletion. Negative beta: proteins were positively associated with a better oxidative capacity; positive beta: proteins were associated with a poorer oxidative capacity.

Demographic characteristics of 165 study participants are displayed in Table 1. Participants were 45% female, in the age range of 22–93 years (average 57.7 ± 20), and mostly Caucasian. Out of the 1301 SOMAmers analyzed, we identified 87 proteins significantly (*p* < 0.01) associated with muscle mitochondrial oxidative capacity (τ_PCr_), adjusting for age, sex, and PCr depletion (Figure 1, Table 2). Sixty proteins were negatively associated with τ_PCr_, therefore positively associated with a better oxidative capacity, while 27 proteins were associated with a poorer oxidative capacity. The top 10 proteins most strongly associated with τ_PCr_ in the first model were endothelial cell-selective adhesion molecule (ESAM), insulin-like growth factor binding protein 3 (IGFBP-3), contactin 2 (CNTN2), p-selectin (coded by the *SELP* gene), proto-oncogene tyrosine-protein kinase Fyn (FYN), lactoperoxidase (PERL), dermatopontin (DERM), C-X-C motif chemokine ligand 16 (CXCL16), Ras-related C3 botulinum toxin substrate 3 (RAC3), and tyrosine-protein kinase Lyn (LYN) (Table 3, Model 1). After adding race and BMI to the multivariable model, 62 proteins were significantly associated with τ_PCr_. The top 10 significant proteins were similar to those resulting from model 1, with the new appearance of kallikrein 11 (coded by the *KLK11* gene), follistatin-related protein 3 (FSTL3), cathepsin F (CATF), and IGFBP-6 (Table 3, Model 2).

**Figure 2:**
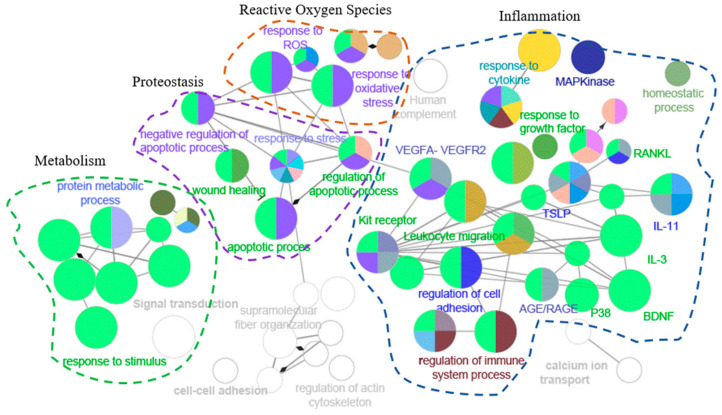
Network visualization of the 87 proteins associated with muscle oxidative capacity. Pathways are functionally grouped and interconnected by edges. Each node is a pathway, and the size of the node represents the significance of the pathway enrichment. The most biologically significant pathways for the purposes of our study are colored, and functionally similar pathways are outlined.

When analyzing the patterns of functional enrichment, many gene ontology (GO) terms were significantly enriched among the 87 proteins significantly correlated with mitochondrial oxidative capacity (Table 4). The 87 significant proteins were enriched for genes in the oxidative stress, inflammation, metabolism regulation, and proteostasis pathways (Figure 2).

## 3. Discussion

In this study we characterized the plasma proteomic profile associated with skeletal muscle oxidative capacity. Using the 1.3 k SOMAscan assay, we found that 87 proteins were associated with mitochondrial oxidative capacity, as measured by ^31^P-MRS on the quadriceps muscle. Three of these proteins (heat shock protein family D member 1, HSP 60, the serine protease HTRA2, and cyclophilin F, coded by the *PPIF* gene) are defined as mitochondrial proteins, and many of the other proteins identified are relevant to mitochondrial functions, or to processes that have been linked with impaired mitochondria. This proteomic signature is reflective of the deranged metabolic mechanisms that are either causes or consequences of impaired mitochondrial function, independent of chronological age. Importantly, although a few proteins found in our analysis are specific to either the muscle (e.g., myostatin) or the blood tissues, most of the proteins are ubiquitous and can be detected in different tissues. From the available data, it is difficult to identify which tissues the plasma proteins represent. Whether the signature identified is a direct marker of muscle function or a marker of a more generalized energetic alteration reflected in plasma proteins cannot be determined.

The most relevant proteins to the association with oxidative capacity are summarized based on our bioinformatic analyses of the enriched processes.

### 3.1. Energy Metabolism

Multiple proteins involved in the regulation of metabolism showed a strong association with muscle oxidative capacity. IGFBP-3 and IGFBP-6 are the carriers of insulin-like growth factor-1 (IGF-1), the primary effector of growth hormone. In addition to its insulin-like functions, IGF-1 stimulates cell growth and proliferation in most tissues of the body and inhibits apoptosis [12]. IGFBP-3 and IGFBP-6 prolong the half-life of IGF-1 and regulate the growth promoting effects of IGF-1, altering its interaction with cell surface receptors. IGFBP-3 also exhibits IGF-independent antiproliferative and apoptotic effects. Several other proteins significantly associated with τ_PCr_ are involved in apoptosis, among which caspase 3 and the protease HTRA2. This finding is particularly interesting because increased apoptosis signaling has been implicated in the pathogenesis of age-related sarcopenia [13].

Decreased plasmatic levels of the adipose-derived hormone leptin were found associated with better oxidative capacity. Leptin is one of the main regulators of feeding and energy balance, connecting changes in energy stores to a set of adaptive physiologic responses. Leptin regulates numerous physiologic processes, among which feeding behavior, metabolism, thermogenesis, immune function, and the neuroendocrine axis [14]. Increased leptin has been related to obesity, inflammation, hypoxia, and has been implicated in ROS generation [15,16]. Any of these mechanisms could underlie the association showed by our data.

Plasma concentrations of the AMP-activated protein kinase (AMPK) a2b2g1 complex were positively associated with better mitochondrial oxidative capacity in this analysis. AMPK is the master sensor of energy metabolism and its activity is principally modulated by the AMP/ATP ratio and, to a lesser extent, by the ADP/ATP ratio, which are direct biomarkers of the status of energy availability. AMPK responds to reduced energy availability by downregulating activities that are energy demanding such as protein and lipid synthesis and cell cycle, and improves energy production through increased catabolism [17]. Moreover, AMPK modulates fundamental mitochondrial processes such as biogenesis, fission, and autophagy, and promotes mitochondrial health [17].

Aldolase A and pyruvate kinase (PKM2) are two key glycolytic enzymes that were found positively associated with oxidative capacity. Other than allowing production of ATP through glycolysis, aldolase A also modulates the myocyte’s shape and contractility, and its absence has been related to metabolic myopathy [18,19]. Aldolase A is prominently expressed in skeletal muscle.

Nicotinamide phosphoribosyltransferase (PBEF, coded by *NAMPT*) catalyzes the rate limiting reaction of the mammalian nicotinamide adenine dinucleotide (NAD^+^) salvage pathway. NAD^+^ is an essential cofactor regulating several metabolic processes such as glycolysis, fatty acid oxidation, the tricarboxylic acid cycle, and oxidative phosphorylation, but also mitochondrial biogenesis [20]. NAD^+^ levels have been implicated in aging, age-related diseases, and longevity [21]. PBEF is released by multiple cell types including myocytes, and it was associated with lower oxidative capacity in our analysis. Arguably, this could reflect either a spilling from damaged muscle cells, or a compensation for a dietary deficiency of NAD^+^ precursors.

Enrichment for the Kit signaling pathways, represented by proteins such as FYN, VAV, LYN, protein kinase C alpha (PKC-A), and proto-oncogene tyrosine-protein kinase Src (SRC), was found. Although it is best known for its role in hematopoietic stem cell differentiation, Kit plays an important role in the regulation of mitochondrial function and energy expenditure [22]. Kit promotes the expression of the peroxisome proliferator-activated receptor-γ (PPARγ) coactivator-1α (PGC-1α), the master regulator of mitochondrial biogenesis and function [23]. PGC-1α has been found to specifically promote mitochondrial biogenesis in skeletal muscle, and its deficiency has been correlated with metabolic derangements and muscle dysfunction [24].

Another pathway significantly enriched was that of the advanced glycation end products (AGEs). AGEs are a heterogeneous group of bioactive molecules formed by the nonenzymatic glycation of proteins, lipids, and nucleic acids. AGEs accumulate with aging in several tissues and contribute to oxidative stress, chronic inflammation, and are implicated in the pathogenesis of cardiovascular diseases and chronic kidney disease [25].

### 3.2. Proteostasis

In this analysis, we found that the plasma levels of several proteins implicated in proteostasis were significantly different across mitochondrial function. Heat shock protein 27 (HSP 27), a low-weight molecular chaperone that maintains denatured proteins in a folding-competent state, in skeletal muscle plays an important role in stress resistance and actin organization [26]. HSP 60 and cyclophilin F participate in mitochondrial import and correct folding of proteins. In addition, cyclophilin F is a major component of the mitochondrial permeability transition pore (MPTP) highly involved in connecting mitochondrial metabolism and apoptosis [27]. Cyclophilin D, coded by *PPID*, is another enzyme assisting and accelerating the correct folding of proteins. HSP 27, HSP 60, cyclophilin F, and cyclophilin D plasma concentrations were positively associated with better oxidative capacity.

DnaJ homolog subfamily B member 1 (DNJB1) interacts with HSP 70 and can stimulate its ATPase activity, facilitating ATP hydrolysis and protein folding. Previous studies found that decreased HSP 70 response was associated with age-related functional impairments in skeletal muscle [28], and that overexpression of HSP 70 in transgenic mice conveyed protection against age-related dysfunction [29], supporting the concept of chaperones as essential molecules to restore the normal cell function after an insult [30]. DNJB1 levels were associated with better mitochondrial oxidative capacity.

Interestingly, levels of two proteins involved in protein degradation and turnover were associated with poorer oxidative capacity: cathepsin F, a major component of the lysosomal system, and ubiquitin-conjugating enzyme E2 G2 (UB2G2), which targets abnormal proteins and catalyzes the attachment of ubiquitin.

### 3.3. Inflammation and Response to Reactive Oxygen Species

A strong connection between mitochondrial impairment and chronic inflammation has been gaining increasing attention [31]. Dysfunctional mitochondria produce an excessive amount of reactive oxygen species (ROS), which trigger inflammation both directly and through oxidative damage to proteins, lipids, and nucleic acids [32]. Furthermore, products of damaged mitochondria released in the extracellular space act as damage-associated molecular pattern (DAMP) agents, activating the immune response [32]. Among the proteins associated with poorer mitochondrial function in our analysis, many were markers of inflammation. Proteins involved in the signaling of cytokines, in chemotaxis, and in the response to oxidative stress, showed a marked prevalence in the relevant clusters identified. Several proteins represented either components or activators of the mitogen-activated protein kinase (MAPK) signal transduction pathway, such as MAPK14, MAPK2, tyrosine-protein kinase Lyn (LYN), sphingosine kinase 1 (Q9NYA1), PKC-A, PKC-B, SLAM family member 5 (SLAF5), and fibroblast growth factor receptor 4 (FGFR4). The MAPK signaling cascade regulates survival and death, proliferation, and differentiation of cells, and is an important activator of the inflammatory response. A strict control is therefore crucial, and its disruption has been linked to the development of many diseases [33].

Many proinflammatory molecules have been identified among the members of the senescence-associated secretory phenotype (SASP). SASP is the secretome of senescent cells that contains hundreds of compounds, some of which have not been yet identified [34]. There is some evidence that the SASP can induce senescence in surrounding cells and cause damage accumulation both in cells and intercellular matrix, events that may contribute to the phenotypes of aging as well as to many chronic diseases [35,36]. It is well known that the energic crisis caused by mitochondrial dysfunction can induce cellular senescence, and the finding that common plasma biomarkers of senescence are dysregulated according to mitochondrial function is not fully surprising [37].

### 3.4. Growth Differentiation Factor 15

Previous studies have proposed growth differentiation factor 15 (GDF15) as a biomarker of mitochondrial dysfunction in aging and several age-related diseases [37]. Increased blood levels of GDF15 have been observed in aging and in mitochondrial disease, and this protein has been related to cardiovascular and brain disease [38,39]. GDF15 has been identified among the molecules expressed by senescent cells which constitute the SASP [34]. Although GDF15 did not appear among the most significant proteins in our analysis, it showed an association (*p* = 0.026) with τ_PCr_ (Model 1); as expected, poor oxidative capacity was associated with higher levels of GDF15 (β coefficient = 0.011).

## 4. Materials and Methods

### 4.1. Participants

This study was conducted in 165 community-dwelling volunteers participating in the Baltimore Longitudinal Study of Aging (BLSA, *N* = 76) and the Genetic and Epigenetic Signatures of Translational Aging Laboratory Testing (GESTALT, *N* = 89) studies.

The BLSA is a prospective open cohort study that has continuously enrolled participants aged 20 and older since 1958. The GESTALT study started in April 2015, aimed at discovering new and sensitive molecular biomarkers of aging in different cell types. Volunteers are eligible to participate in BLSA and GESTALT if they meet strict healthy inclusion criteria, where participants are free of major pathologies (with the exception of controlled hypertension) as well as functional and cognitive impairments at enrollment, and are followed for life regardless of changes in health and functional status (Supplementary Appendix A). This analysis was performed on samples collected during visits in which participants met the inclusion criteria.

All assessments, which took place at the Clinical Research Unit of the Intramural Research Program of the National Institute on Aging, National Institutes of Health (NIH) during a 2.5–3.5-day visit, were performed by certified nurse practitioners and certified technicians according to standardized procedures. The protocol for both studies was approved by the NIH Intramural Institutional Review Board (BLSA (03AG0325) and GESTALT (15AG0063) were approved on 12 May 2020). After receiving detailed descriptions of the procedures at every visit, all subjects provided written informed consent.

Demographic and health characteristics were assessed either through self-report questionnaires or using standard criteria and algorithms [40]. Body weight was measured in kilograms using a calibrated scale to the nearest 0.1 kg. Body height was measured in centimeters by a stadiometer to the nearest 0.1 cm [41]. Body mass index (BMI) was calculated by dividing body weight by the square of height in meters.

### 4.2. Proteomic Assessment

Plasma proteins were measured using overnight fasted plasma that was collected at a resting state and subsequently stored at −80 °C. Discovery proteomics was performed using the 1.3 k SOMAscan Assay (SomaLogic, Inc.; Boulder, CO, USA) at the Trans-NIH Center for Human Immunology and Autoimmunity, and Inflammation (CHI), National Institute of Allergy and Infectious Disease, National Institutes of Health (Bethesda, MD, USA). SOMAmer reagents are individually generated via an iterative process called SELEX (Systematic Evolution of Ligands by EXponential enrichment), which consists of affinity selection cycles aimed at increasing the specificity and avidity of oligomers to a target protein epitope. As a result, SOMAmer reagents are designed to be highly specific and sensitive. For a quantitative assessment of variability in the SOMAscan assay, see [42,43]. A discussion of caveats and limitations is provided in [44].

Of the 1322 SOMAmer reagents included in this version of the kit, 12 hybridization controls, four viral proteins (HPV type 16, HPV type 18, isolate BEN, isolate LW123), and five SOMAmers that were reported to be nonspecific (P05186; ALPL, P09871; C1S, Q14126; DSG2, Q93038; TNFRSF25, Q9NQC3; RTN4) were removed, leaving 1301 SOMAmer reagents for the final analysis. There are 46 SOMAmer reagents that are documented to target multicomplex proteins of two or more unique proteins (UniProt IDs). Conversely, there are 49 UniProt IDs that are measured by more than one SOMAmer reagent. The full list of SOMAmer reagents and their protein targets is provided as Supplementary Information. Thus, the 1301 SOMAmer reagents collectively target 1297 UniProt IDs. Of note, there are four proteins in the final protein panel that are rat homologues (P05413; FABP3, P48788; TINNI2, P19429; TINNI3, P01160; NPPA) of human proteins.

The experimental process for proteomic assessment and data normalization has been previously described [42]. The data reported are SOMAmer reagent abundance in relative fluorescence units (RFU). The abundance of the SOMAmer reagent represents a surrogate of protein concentration in the plasma sample.

Data normalization was conducted in three stages. First, hybridization control normalization removed individual sample variance on the basis of signaling differences between microarray or Agilent scanner. Second, median signal normalization removed intersample differences within a plate due to technical differences such as pipetting variation. Last, calibration normalization removed variance across assay runs. Furthermore, there was an additional interplate normalization process that utilized a CHI calibrator of pooled plasma from healthy subjects that allowed normalization across all experiments conducted at the CHI laboratory [42]. An interactive Shiny web tool was used during the CHI QC process [45].

### 4.3. Phosphorus Magnetic Resonance Spectroscopy

**Figure 3:**
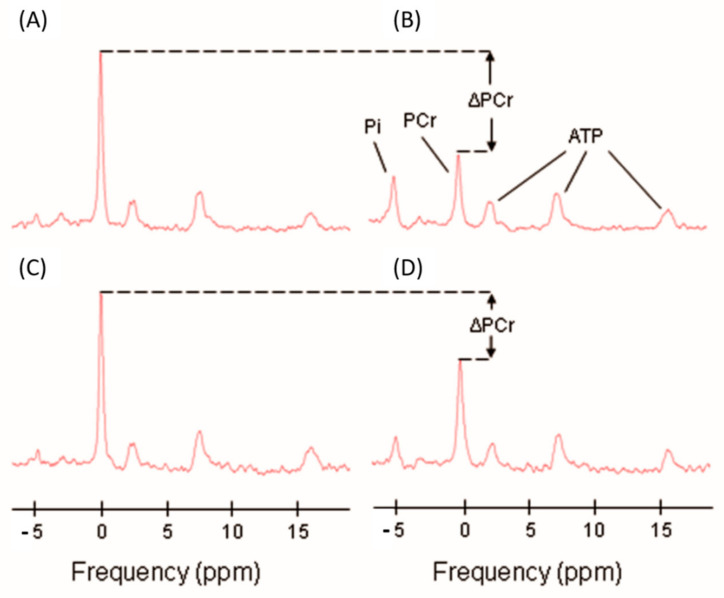
Representative ^31^P spectra with inorganic phosphate (Pi), phosphocreatine (PCr), and ATP resonances indicated in panel (**B**). Panels (**A**,**B**) show examples of baseline and postexercise spectra of a typical level of exercise induced PCr depletion (ΔPCr). Panels (**C**,**D**) represent examples of baseline and postexercise spectra with a minimally acceptable level of PCr depletion (ΔPCr = 33%) [7].

Using a 3T MR scanner (Achieva, Philips Healthcare, Andover, MA, USA), in vivo ^31^P-MRS measurements of the concentrations of the phosphorus-containing metabolites phosphocreatine (PCr), inorganic phosphate (Pi), and ATP were obtained from the vastus lateralis muscle of the left thigh, following a standardized protocol described previously [7,46]. Participants were positioned supine on the bed of the scanner, with a foam wedge placed underneath the knee to induce slight flexion, and with ankles, thighs, and hips secured with straps to reduce movement during exercise. Participants were required to perform a ballistic knee extension exercise inside the magnet with their left leg, while a resistance was added by foam pads placed above the left leg in order to enhance the intensity of the exercise. A series of pulse-acquire ^31^P spectra were obtained before, during, and after the exercise, which had an average duration of 30 s, with a repetition time of 1.5 s, using a 10-cm ^31^P-tuned surface coil (PulseTeq, Surrey, UK) fastened above the left thigh. An example of the acquired spectra is represented in Figure 3 [7]. Signals were averaged over four successive acquisitions for signal-to-noise ratio enhancement, so that the data consisted of 75 spectra obtained with a temporal resolution of 6 s. The duration of exercise was optimized by consistently requiring a depletion in PCr of 50–67% relative to initial baseline values, in order to standardize the measure of oxidative function across different subjects and to provide sufficient dynamic range to fit the PCr recovery curve. Whenever PCr depletion did not reach the threshold of 33%, data collected were excluded from further analysis. If intramuscular acidosis, defined as intracellular pH lower than 6.8, was detected at the end of the exercise, the test was repeated at a lower intensity after waiting for the participant to return to a resting condition [47]. The pH was determined according to the chemical shift of Pi relative to PCr [48]. Spectra were processed with jMRUI software (version 5.2, MRUI Consortium), and metabolite concentrations were calculated by nonlinear least squares fitting implemented through AMARES [49,50].

Post-exercise PCr recovery rates were calculated by fitting time-dependent changes in PCr peak area to the monoexponential recovery function:

$$\mathrm{PCr}\left(t\right)\;=\mathrm{PCr}\left(0\right)\;+\;\Delta \mathrm{PCr}\times (1-\exp\left(-\frac{t}{\tau_{\mathrm{PCr}}})\;\right)$$

where PCr(0) is the end-of-exercise PCr signal area (i.e., the PCr signal area at the beginning of the recovery period), ΔPCr is the decrease in signal area from its pre-exercise baseline value, averaged from the multiple baseline scans, to PCr(0) resulting from in-magnet exercise, and τ_PCr_ is the PCr exponential recovery time constant, measured in seconds [7]. This time constant is inversely proportional to the maximum in vivo oxidative capacity of skeletal muscle, with longer τ_PCr_ reflecting slower recovery and therefore lower oxidative capacity [51]. Since the energy demands during post-exercise PCr resynthesis are minimal, 1/τ_PCr_ reflects the maximum mitochondrial ATP production rate [7,52,53,54]. ATPmax was finally estimated as [PCr_baseline_]∗(1/τ_PCr_) [55].

### 4.4. Statistical Analysis

Protein RFU values were converted to a z-score after natural log-transformation. Association of each protein with mitochondrial oxidative capacity (τ_PCr_) was assessed using linear regression models adjusted for age, sex, and amount of PCr depletion. A second model was examined with further adjustments for race (white, black, other), and BMI. The analyses were performed using RStudio (v. 1.2.1335). A nominal *p* value of 0.01 was considered statistically significant.

### 4.5. Enrichment Analysis

To evaluate whether among the proteins significantly correlated with τ_PCr_ appeared to be enrichment of specific biological processes or molecular functions, a gene enrichment analysis was run on the 87 plasma proteins significantly associated with τ_PCr_. For this purpose, the bioinformatic tool ClueGO was used [56]. ClueGO permitted the identification of functional gene ontologies (GO) and pathways associated with the most significant proteins in our analysis. To visualize the enriched pathways, the Cytoscape (v. 3.8.0) plug-in was used. The resulting enriched pathways were displayed as a network, in which each pathway was represented as a node and the edges connected similar pathways; pathways were functionally grouped, and the similarity between pathways was determined by kappa statistics [56]. Enrichment significance was represented by node size, and node colors were used to differentiate pathway clusters. The relevant pathways were filtered for > 4 genes after Bonferroni correction.

## 5. Limitations and Conclusions

The SOMAscan platform used in this study assesses 1301 proteins, which represent only a fraction of the proteins that are potentially important for aging or mitochondrial function. Furthermore, the SOMAscan technology does not provide an absolute measure of protein abundance, which makes it difficult to compare associations with mitochondrial function across proteins. In addition, while aptamers are designed to detect proteins in their native conformation, there is also a possibility of cross-reactivity between similar proteins, which can limit the accuracy of SOMAscan for proteins with high sequence homology [57].

Importantly, the list of proteins identified do not completely reflect those of the proteins that have been found to be associated with aging. This could be due to the fact that while mitochondrial oxidative capacity declines with aging, the rate of decline is highly heterogeneous across individuals, probably because of the effect of genetic heterogeneity and subclinical and clinical pathology. Due to the limited sample size and number of proteins studied we could not determine an exhaustive proteomic signature and fully understand what biological pathways are shared between aging and mitochondrial function.

The inclusion criteria of BLSA and GESTALT ensure that study participants are exceptionally healthy, and therefore our findings may not be generalizable to a population affected by substantial morbidity or disability. In addition, the cross-sectional nature of our analysis makes it impossible to discriminate whether the proteins associated with differential mitochondrial function in this study represent causes or consequences. Further longitudinal studies should better disentangle the mechanisms underlying the associations identified.

Finally, although ^31^P-MRS has been long considered a procedure able to provide a measure of mitochondrial oxidative capacity generalizable to many tissues, the measure of mitochondrial oxidative capacity used in this study is relative to the quadriceps muscle and it is possible that the energetic status of this muscle may not be representative of the energetic status in other muscle groups or tissues. Hence, the interference of other tissues may have reduced the signal-to-noise ratio weakening the results of this study.

In conclusion, mitochondrial oxidative capacity of skeletal muscle was associated with specific clusters of plasma proteins in this study, mainly representing the following pathways: homeostasis of energy metabolism, protein turnover, and inflammation. These findings need to be replicated in an independent population, possibly with longitudinal data and alternative measurements of mitochondrial function, before the results can be used to develop a clinical tool to assess mitochondrial function using the blood proteome.

## Figures and Tables

**Table 1:** Demographic and health characteristics of 165 study participants.

Characteristic		Mean (SD, min, max) or %
Age, years		57.7 (20.0, 22, 93)
Race, %	White	77.6
	Black	15.8
	Other	6.6
Sex, % female		45.4
BMI, kg/m^2^		25.9 (3.2, 18.9, 39.7)
τ_PCr_, s		47.5 (11.3, 23.1, 78.8)
ATP max		0.41 (0.14, 0.06, 0.71)
pH at baseline		7.07 (0.03, 6.97, 7.14)

**Table 2:** Comprehensive list of 87 SOMAmers associated with τ_PCr_, an inverse measure of mitochondrial oxidative capacity, adjusting for age, sex, and amount of phosphocreatine depletion (*p* < 0.01).

SomaId	GeneID	Target	β Coefficient	SE	*p*
SL005160	*ESAM*	ESAM	−0.036	0.010	0.0003
SL000045	*IGFBP3*	IGFBP-3	−0.027	0.007	0.0005
SL008623	*CNTN2*	CNTN2	0.032	0.009	0.0005
SL000560	*SELP*	P-Selectin	−0.030	0.008	0.0006
SL006913	*FYN*	FYN	−0.030	0.009	0.0009
SL007153	*LPO*	PERL	0.031	0.009	0.0009
SL008178	*DPT*	DERM	0.027	0.008	0.0009
SL004016	*CXCL16*	CXCL16, soluble	0.023	0.009	0.001
SL007310	*RAC3*	RAC3	−0.03	0.009	0.001
SL006917	*LYN*	LYN	−0.03	0.009	0.002
SL002763	*KLK11*	Kallikrein 11	0.033	0.010	0.002
SL008381	*CTSF*	CATF	0.029	0.009	0.002
SL009324	*FSTL3*	FSTL3	0.023	0.007	0.002
SL001947	*MIA*	MIA	0.029	0.009	0.002
SL014488	*VAV1*	VAV	−0.029	0.009	0.002
SL010518	*TEC*	TEC	−0.028	0.009	0.002
SL000448	*HSPB1*	HSP 27	−0.030	0.010	0.002
SL005588	*FER*	FER	−0.028	0.009	0.002
SL004920	*CFL1*	Cofilin-1	−0.027	0.009	0.002
SL005793	*PPIF*	Cyclophilin F	−0.028	0.009	0.002
SL013488	*CLEC1B*	CLC1B	−0.028	0.009	0.003
SL003792	*GRB2*	GRB2 adapter protein	−0.028	0.009	0.003
SL008588	*CD84*	SLAF5	−0.027	0.009	0.003
SL006372	*YES1*	YES	−0.030	0.010	0.003
SL005266	*SNCA*	a-Synuclein	−0.028	0.009	0.003
SL010516	*SRC*	SRCN1	−0.029	0.009	0.003
SL004101	*SMAD2*	SMAD2	−0.027	0.009	0.003
SL011211	*EIF4G2*	IF4G2	−0.027	0.009	0.003
SL010500	*LYN*	LYNB	−0.028	0.009	0.003
SL000551	*PRKCA*	PKC-A	−0.028	0.009	0.003
SL010374	*METAP1*	METAP1	−0.027	0.009	0.003
SL005172	*IGFBP6*	IGFBP-6	0.023	0.008	0.004
SL003739	*TNFRSF6B*	DcR3	0.028	0.009	0.004
SL010927	*KPNB1*	IMB1	−0.026	0.009	0.004
SL006088	*SPHK1*	Sphingosine kinase 1	−0.027	0.009	0.004
SL002922	*ICAM1*	sICAM-1	0.023	0.008	0.004
SL006998	*PDPK1*	PDPK1	−0.026	0.009	0.004
SL004869	*CA13*	Carbonic anhydrase XIII	−0.026	0.009	0.004
SL004914	*PPA1*	PPase	−0.027	0.009	0.004
SL014469	*SHC1*	SHC1	−0.025	0.008	0.004
SL002823	*SELL*	sL-Selectin	−0.024	0.008	0.004
SL008759	*GP6*	GPVI	−0.026	0.009	0.005
SL004921	*NME2*	NDP kinase B	−0.026	0.009	0.005
SL005687	*EIF5A*	eIF-5A-1	−0.026	0.009	0.005
SL004339	*FGF5*	FGF-5	0.026	0.009	0.005
SL000553	*PRKCB*	PKC-B-II	−0.027	0.009	0.005
SL000498	*LEP*	Leptin	0.022	0.008	0.005
SL011405	*PDE5A*	PDE5A	−0.026	0.009	0.006
SL000449	*DNAJB1*	HSP 40	−0.025	0.009	0.006
SL004760	*PAFAH1B2*	PAFAH beta subunit	−0.026	0.009	0.006
SL005688	*YWHAZ*	14-3-3 protein zeta/delta	−0.027	0.009	0.006
SL016549	*PRKAA2 PRKAB2 PRKAG1*	AMPK a2b2g1	−0.025	0.009	0.006
SL000337	*CAPN1 CAPNS1*	Calpain I	−0.026	0.009	0.006
SL018946	*UBE2G2*	UB2G2	0.028	0.010	0.006
SL006920	*MAPK14*	MAPK14	−0.025	0.009	0.006
SL004704	*COMMD7*	COMMD7	−0.026	0.009	0.006
SL007221	*STAT3*	STAT3	−0.026	0.009	0.006
SL004676	*IGFBP5*	IGFBP-5	0.027	0.010	0.006
SL003646	*TPM4*	Tropomyosin 4	−0.027	0.010	0.006
SL010373	*FCAR*	FCAR	0.026	0.009	0.007
SL003685	*NAMPT*	PBEF	0.027	0.010	0.007
SL008611	*NAAA*	ASAHL	0.025	0.009	0.007
SL004757	*VTA1*	DRG-1	−0.025	0.009	0.007
SL004860	*HTRA2*	HTRA2	−0.025	0.009	0.007
SL003711	*CASP3*	Caspase-3	−0.025	0.009	0.007
SL003655	*TKT*	Transketolase	−0.025	0.009	0.007
SL017188	*GSK3A GSK3B*	GSK-3 alpha/beta	−0.025	0.009	0.007
SL005372	*SNX4*	Sorting nexin 4	−0.025	0.009	0.008
SL004536	*HAMP*	LEAP-1	0.025	0.009	0.008
SL003690	*TNFRSF11A*	RANK	0.025	0.009	0.009
SL010503	*MAPKAPK2*	MAPK2	−0.024	0.009	0.008
SL010521	*BTK*	BTK	−0.025	0.009	0.008
SL005084	*POSTN*	Periostin	0.024	0.009	0.008
SL004357	*MSTN*	Myostatin	0.022	0.008	0.008
SL004910	*ALDOA*	aldolase A	−0.023	0.009	0.008
SL002036	*FGFR4*	FGFR4	0.027	0.010	0.008
SL000450	*HSPD1*	HSP 60	−0.025	0.009	0.009
SL011630	*SEZ6L2*	SE6L2	−0.024	0.009	0.009
SL002650	*PKM2*	M2-PK	−0.025	0.009	0.009
SL004924	*EIF4H*	eIF-4H	−0.023	0.009	0.009
SL010493	*CAMK2D*	CAMK2D	−0.024	0.009	0.009
SL004139	*EFNA3*	Ephrin-A3	0.025	0.009	0.009
SL004672	*TNFRSF17*	BCMA	0.024	0.009	0.009
SL007373	*PPID*	PPID	−0.024	0.009	0.009
SL011404	*PDE4D*	PDE4D	0.026	0.010	0.009
SL010491	*CAMK2A*	CAMK2A	−0.023	0.009	0.009
SL000565	*REN*	Renin	0.025	0.009	0.009

**Table 3:** Top-10 most significant SOMAmers associated with τ_PCr_, an inverse measure of mitochondrial oxidative capacity, adjusting for age, sex, and amount of phosphocreatine depletion (Model 1); top-10 most significant SOMAmers associated with τ_PCr_ adjusting for age, sex, phosphocreatine depletion, BMI, and race (Model 2).

Model 1					
SomaId	GeneID	Target	β Coefficient	SE	*p*
SL005160	*ESAM*	ESAM	−0.036	0.010	0.0003
SL000045	*IGFBP3*	IGFBP-3	−0.027	0.007	0.0005
SL008623	*CNTN2*	CNTN2	0.032	0.009	0.0005
SL000560	*SELP*	P-Selectin	−0.030	0.008	0.0006
SL006913	*FYN*	FYN	−0.030	0.009	0.0009
SL007153	*LPO*	PERL	0.031	0.009	0.0009
SL008178	*DPT*	DERM	0.027	0.008	0.001
SL004016	*CXCL16*	CXCL16, soluble	0.029	0.009	0.001
SL007310	*RAC3*	RAC3	−0.029	0.009	0.001
SL006917	*LYN*	LYN	−0.030	0.009	0.002
**Model 2**					
**SomaId**	**GeneID**	**Target**	**β Coefficient**	**SE**	***p***
SL002763	*KLK11*	Kallikrein 11	0.034	0.010	0.0006
SL000560	*SELP*	P-Selectin	−0.031	0.009	0.0006
SL006913	*FYN*	FYN	−0.030	0.009	0.001
SL009324	*FSTL3*	FSTL3	0.023	0.007	0.001
SL008623	*CNTN2*	CNTN2	0.030	0.009	0.002
SL000045	*IGFBP3*	IGFBP-3	−0.025	0.008	0.002
SL005160	*ESAM*	ESAM	−0.032	0.010	0.002
SL007310	*RAC3*	RAC3	−0.029	0.009	0.002
SL008381	*CTSF*	CATF	0.028	0.009	0.003
SL005172	*IGFBP6*	IGFBP-6	0.025	0.008	0.003

**Table 4:** Significantly enriched gene ontology (GO) pathways in the association with muscle mitochondrial oxidative capacity, adjusting for age and sex.

GO id	GO Term	N. Genes	Associated Genes Found
GO:0007165	Signal transduction	59	*BTK*, *CAMK2A*, *CAMK2D*, *CASP3*, *CFL1*, *CLEC1B*, *CNTN2*, *COMMD7*, *CXCL16*, *EFNA3*, *FER*, *FGF5*, *FGFR4*, *FSTL3*, *FYN*, *GP6*, *GRB2*, *HAMP*, *HSPB1*, *HSPD1*, *HTRA2*, *ICAM1*, *IGFBP3*, *IGFBP5*, *IGFBP6*, *KPNB1*, *LEP, LYN*, *MAPK14*, *MAPKAPK2*, *METAP1*, *MIA*, *MSTN*, *NAMPT*, *NME2*, *PDE4D, PDE5A*, *PDPK1*, *POSTN*, *PPIF*, *PRKCA*, *PRKCB*, *RAC3*, *REN*, *SELP*, *SEZ6L2*, *SHC1*, *SMAD2*, *SNCA*, *SPHK1, SRC*, *STAT3*, *TEC*, *TNFRSF11A*, *TNFRSF17*, *TNFRSF6B*, *VAV1*, *YES1*, *YWHAZ*
GO:0010033	Response to organic substance	50	*BTK, CAMK2A, CAMK2D, CASP3, CFL1, COMMD7, CXCL16, DNAJB1, FER, FGF5, FGFR4, FSTL3, FYN, GRB2, HAMP, HSPB1, HSPD1, HTRA2, ICAM1, IGFBP5, LEP, LYN, MAPK14, MAPKAPK2, MSTN, NAMPT, NME2, PDE4D, PDPK1, PKM, POSTN, PRKCA, PRKCB, REN, SELL, SELP, SHC1, SMAD2, SNCA, SPHK1, SRC, STAT3, TEC, TNFRSF11A, TNFRSF17, TNFRSF6B*, *UBE2G2*, *VAV1*, *YES1, YWHAZ*
GO:0071310	Cellular response to organic substance	46	*BTK, CAMK2A, CAMK2D, CASP3, CFL1, COMMD7, FER, FGF5, FGFR4, FSTL3, FYN, GRB2, HAMP, HSPB1, HSPD1, HTRA2, ICAM1, IGFBP5, LEP, LYN, MAPK14, MAPKAPK2, MSTN, NAMPT, NME2, PDE4D, PDPK1, PKM, POSTN, PRKCA, PRKCB, REN, SHC1, SMAD2, SNCA, SPHK1, SRC, STAT3, TEC, TNFRSF11A, TNFRSF17, TNFRSF6B, UBE2G2, VAV1, YES1, YWHAZ*
GO:0051239	Regulation of multicellular organismal process	41	*BTK, CAMK2A, CAMK2D, CD84, CFL1, CNTN2, EFNA3, EIF4G2, FSTL3, FYN, HAMP, HSPB1, HSPD1, HTRA2, ICAM1, IGFBP5, LEP, LYN, MAPK14, MAPKAPK2, MSTN, NME2, PDE4D, PDE5A, PDPK1, PKM, POSTN, PRKCA, PRKCB, RAC3, REN, SELP, SMAD2, SNCA, SNX4, SPHK1, SRC, STAT3, TEC, TNFRSF11A, YWHAZ*
GO:0032268	Regulation of cellular protein metabolic process	38	*CAMK2D, CASP3, CNTN2, EFNA3, EIF4G2, EIF4H, EIF5A, FER, FGFR4, FYN, GRB2, HSPB1, HSPD1, HTRA2, ICAM1, IGFBP3, IGFBP5, LEP, LYN, MAPK14, MAPKAPK2, METAP1, MSTN, PDE4D, PDE5A, PDPK1, PRKCA, REN, SHC1, SNCA, SPHK1, SRC, STAT3, TEC, TNFRSF11A, UBE2G2, YES1, YWHAZ*
GO:1901700	Response to oxygen-containing compound	37	*BTK, CAMK2A, CASP3, CFL1, FER, FYN, GRB2, HAMP, HSPD1, HTRA2, ICAM1, IGFBP5, LEP, LYN, MAPK14, MAPKAPK2, MSTN, NAMPT, NME2, PDE4D, PDPK1, PKM, POSTN, PPIF, PRKCA, PRKCB, REN, SELL, SELP, SHC1, SMAD2, SNCA, SPHK1, SRC, STAT3, TNFRSF11A, YES1*
GO:0032879	Regulation of localization	36	*CAMK2A, CAMK2D, CD84, CXCL16, FER, FYN, HAMP, HSPB1, HSPD1, HTRA2, ICAM1, IGFBP3, IGFBP5, LEP, LYN, MAPK14, METAP1, MSTN, PDE4D, PDPK1, POSTN, PPID, PPIF, PRKCA, PRKCB, REN, SELP, SNCA, SNX4, SPHK1, SRC, STAT3, TNFRSF11A, UBE2G2, YES1, YWHAZ*
GO:0051128	Regulation of cellular component organization	32	*CAMK2D, CFL1, CNTN2, CXCL16, DNAJB1, EIF4G2, EIF5A, ESAM, FER, FGFR4, FYN, GRB2, HAMP, HTRA2, ICAM1, IGFBP3, IGFBP5, LYN, MAPK14, METAP1, MSTN, NME2, PPIF, PRKCA, PRKCB, RAC3, SELP, SNCA, SPHK1, SRC, VTA1, YWHAZ*
GO:0034097	Response to cytokine	31	*BTK, CAMK2A, CAMK2D, CASP3, CFL1, COMMD7, CXCL16, FER, FYN, GRB2, HAMP, HSPD1, HTRA2, ICAM1, LEP, MAPK14, MAPKAPK2, POSTN, PRKCA, SHC1, SNCA, SPHK1, SRC, STAT3, TEC, TNFRSF11A, TNFRSF17, TNFRSF6B, UBE2G2, VAV1, YWHAZ*
GO:0012501	Programmed cell death	31	*BTK, CAMK2A, CAMK2D, CASP3, CFL1, EIF5A, FYN, HSPB1, HSPD1, HTRA2, ICAM1, IGFBP3, KPNB1, LEP, LYN, MAPK14, NME2, PDPK1, PKM, PPID, PPIF, PRKCA, PRKCB, SHC1, SNCA, SPHK1, SRC, TNFRSF11A, TNFRSF6B, VAV1, YWHAZ*
GO:0006915	Apoptotic process	29	*BTK, CAMK2A, CAMK2D, CASP3, CFL1, EIF5A, FYN, HSPB1, HSPD1, HTRA2, ICAM1, IGFBP3, KPNB1, LEP, LYN, MAPK14, NME2, PDPK1, PPID, PPIF, PRKCA, PRKCB, SHC1, SNCA, SPHK1, SRC, TNFRSF6B, VAV1, YWHAZ*
GO:0048468	Cell development	29	*BTK, CAMK2A, CASP3, CFL1, CLEC1B, CNTN2, EFNA3, EIF4G2, FER, FYN, GRB2, HAMP, HTRA2, ICAM1, LEP, LYN, METAP1, NME2, PDE4D, PDE5A, PDPK1, POSTN, PRKCA, RAC3, REN, SHC1, SRC, STAT3, YWHAZ*
GO:0045321	Leukocyte activation	29	*ALDOA, BTK, CASP3, CD84, FCAR, FER, FYN, GRB2, HSPD1, ICAM1, KPNB1, LEP, LYN, MAPK14, NAMPT, NME2, PAFAH1B2, PDE5A, PDPK1, PKM, PRKCB, SELL, SNCA, SNX4, SPHK1, SRC, STAT3, VAV1, YES1*
GO:0032940	Secretion by cell	28	*ALDOA, BTK, CAMK2A, CD84, FCAR, FER, HSPD1, KPNB1, LEP, LYN, MAPK14, NAAA, NME2, PAFAH1B2, PDPK1, PKM, POSTN, PPID, PRKCA, PRKCB, REN, SELL, SELP, SMAD2, SNCA, SNX4, SRC, TNFRSF11A*
GO:0042592	Homeostatic process	27	*ALDOA, CAMK2D, CASP3, ESAM, FGFR4, FYN, HAMP, HSPB1, ICAM1, IGFBP5, LEP, LPO, LYN, MAPK14, METAP1, MSTN, NME2, PDE4D, PDPK1, PRKCA, PRKCB, RAC3, SNCA, SRC, STAT3, TNFRSF11A, TNFRSF17*
GO:0006952	Defense response	27	*BTK, CAMK2A, CAMK2D, CD84, CLEC1B, CXCL16, FYN, HAMP, HSPD1, ICAM1, LEP, LPO, LYN, MAPK14, MAPKAPK2, NAMPT, PDPK1, PRKCA, SELP, SHC1, SNCA, SNX4, SPHK1, SRC, STAT3, TNFRSF11A, VAV1*
GO:0033554	Cellular response to stress	27	*BTK, CAMK2A, CAMK2D, CASP3, DNAJB1, FER, FYN, GRB2, HSPB1, HSPD1, HTRA2, ICAM1, LEP, LYN, MAPK14, MAPKAPK2, NAMPT, NME2, PDPK1, PPIF, PRKCA, SHC1, SNCA, SPHK1, SRC, TNFRSF11A, UBE2G2*
GO:0042127	Regulation of cell population proliferation	27	*BTK, CASP3, DPT, EIF5A, FER, FGF5, FGFR4, FYN, IGFBP3, IGFBP5, IGFBP6, LEP, LYN, MAPK14, MSTN, NAMPT, NME2, PDE5A, PDPK1, PRKCA, SHC1, SMAD2, SPHK1, SRC, STAT3, TNFRSF11A, YES1*
GO:0010243	Response to organonitrogen compound	27	*CAMK2A, CASP3, CFL1, FER, FYN, GRB2, HSPD1, ICAM1, IGFBP5, LEP, LYN, MAPK14, MSTN, NAMPT, NME2, PDE4D, PDPK1, PKM, PRKCA, PRKCB, REN, SELL, SHC1, SNCA, SRC, STAT3, UBE2G2*
GO:0042981	Regulation of apoptotic process	25	*BTK, CAMK2A, CAMK2D, CASP3, CFL1, FYN, HSPB1, HSPD1, HTRA2, ICAM1, IGFBP3, LEP, LYN, NME2, PDPK1, PPID, PPIF, PRKCA, SHC1, SNCA, SPHK1, SRC, TNFRSF6B, VAV1, YWHAZ*
GO:0007169	Transmembrane receptor protein tyrosine kinase signaling pathway	25	*CASP3, EFNA3, FER, FGF5, FGFR4, FYN, GRB2, HSPB1, IGFBP3, IGFBP5, IGFBP6, LEP, LYN, MAPK14, MAPKAPK2, MSTN, PDPK1, PRKCA, PRKCB, SHC1, SNCA, SRC, STAT3, VAV1, YES1*
GO:0048585	Negative regulation of response to stimulus	25	*CD84, FER, FSTL3, FYN, GRB2, HSPB1, HTRA2, ICAM1, IGFBP3, IGFBP5, IGFBP6, LEP, LYN, MAPK14, MSTN, NAMPT, PDE4D, PDPK1, PPIF, PRKCA, PRKCB, SMAD2, SNCA, SRC, UBE2G2*
GO:0080134	Regulation of response to stress	25	*BTK, CAMK2A, CAMK2D, DNAJB1, FYN, HSPB1, HSPD1, HTRA2, LEP, LYN, MAPK14, MAPKAPK2, NAMPT, PDPK1, PRKCA, SELP, SNCA, SNX4, SPHK1, SRC, STAT3, TEC, TNFRSF11A, UBE2G2, VAV1*
GO:0098609	Cell-cell adhesion	24	*CASP3, CD84, CNTN2, ESAM, FER, FSTL3, FYN, GRB2, HSPB1, HSPD1, ICAM1, LEP, LYN, MAPK14, METAP1, PDE5A, PDPK1, PRKCA, SELL, SELP, SHC1, SRC, VAV1, YES1*
GO:0033993	Response to lipid	23	*CASP3, FER, HAMP, HSPD1, HTRA2, ICAM1, LEP, LYN, MAPK14, MAPKAPK2, MSTN, NME2, PDE4D, POSTN, PRKCA, REN, SELP, SMAD2, SNCA, SRC, STAT3, TNFRSF11A, YES1*
GO:0050776	Regulation of immune response	22	*BTK, CD84, CLEC1B, FER, FYN, GRB2, HSPD1, ICAM1, LEP, LYN, MAPK14, PDE4D, PDPK1, PRKCA, PRKCB, SELL, SHC1, SNX4, SRC, TEC, VAV1, YES1*
GO:0070848	Response to growth factor	20	*CASP3, FER, FGF5, FGFR4, FSTL3, FYN, GRB2, HSPB1, HTRA2, MAPK14, MAPKAPK2, MSTN, PDPK1, POSTN, PRKCB, SHC1, SMAD2, SPHK1, SRC, YES1*
GO:0050900	Leukocyte migration	20	*CD84, CXCL16, ESAM, FER, FYN, GP6, GRB2, ICAM1, LEP, LYN, MAPK14, MSTN, PDE4D, SELL, SELP, SHC1, SRC, TNFRSF11A, VAV1, YES1*
GO:0019221	Cytokine-mediated signaling pathway	20	*CAMK2A, CAMK2D, CASP3, CFL1, COMMD7, FER, FYN, GRB2, ICAM1, LEP, PRKCA, SHC1, SPHK1, STAT3, TEC, TNFRSF11A, TNFRSF17, TNFRSF6B, VAV1, YWHAZ*
GO:0042060	Wound healing	19	*CASP3, CLEC1B, FYN, GP6, HSPB1, LYN, METAP1, MSTN, PDPK1, PKM, POSTN, PRKCA, PRKCB, SELP, SMAD2, SRC, TEC, VAV1, YWHAZ*
GO:0022603	Regulation of anatomical structure morphogenesis	19	*ALDOA, CFL1, CNTN2, EFNA3, EIF4G2, FYN, HSPB1, ICAM1, LEP, MAPK14, PDPK1, PKM, POSTN, PRKCA, PRKCB, RAC3, SPHK1, SRC, STAT3*
GO:0000902	Cell morphogenesis	18	*ALDOA, CASP3, CFL1, CLEC1B, CNTN2, EFNA3, EIF4G2, FER, FYN, GRB2, ICAM1, MAPK14, METAP1, POSTN, PRKCA, RAC3, SHC1, SRC*
GO:0035295	Tube development	18	*CASP3, CFL1, EFNA3, FSTL3, HSPB1, IGFBP5, LEP, MAPK14, PDPK1, PKM, PRKCA, PRKCB, SHC1, SMAD2, SPHK1, SRC, STAT3, YWHAZ*
WP:3888	VEGFA-VEGFR2 signaling pathway	17	*ALDOA, CFL1, EIF4G2, FYN, GRB2, HSPB1, ICAM1, IGFBP3, MAPK14, MAPKAPK2, PDPK1, PRKCA, PRKCB, SHC1, SRC, STAT3, TKT*
GO:1903530	Regulation of secretion by cell	17	*CAMK2A, CD84, FER, HSPD1, LEP, LYN, MAPK14, PDPK1, POSTN, PPID, PRKCA, PRKCB, REN, SNCA, SNX4, SRC, TNFRSF11A*
GO:0006935	Chemotaxis	16	*CNTN2, CXCL16, EFNA3, FER, FYN, GRB2, HSPB1, LYN, MAPK14, MSTN, PDE4D, PRKCA, SHC1, SRC, TNFRSF11A, VAV1*
GO:0043408	Regulation of MAPK cascade	16	*FGFR4, GRB2, ICAM1, IGFBP3, LEP, LYN, MAPK14, MAPKAPK2, PDE5A, PRKCA, REN, SHC1, SPHK1, SRC, TNFRSF11A, YWHAZ*
GO:0097435	Supramolecular fiber organization	15	*ALDOA, CFL1, DPT, ESAM, FER, FYN, GRB2, ICAM1, KPNB1, METAP1, RAC3, SNCA, SRC, TPM4, VTA1*
GO:0016032	Viral process	15	*CFL1, CLEC1B, EIF4H, FYN, GRB2, HSPB1, HSPD1, ICAM1, KPNB1, LYN, PPID, SHC1, SRC, STAT3, VTA1*
GO:0019725	Cellular homeostasis	15	*ALDOA, CAMK2D, FYN, HAMP, ICAM1, LPO, LYN, METAP1, MSTN, NME2, PDE4D, PDPK1, PRKCA, PRKCB, SNCA*
GO:0009636	Response to toxic substance	15	*CASP3, FYN, HAMP, HSPD1, HTRA2, ICAM1, LEP, LPO, LYN, MSTN, PPIF, SNCA, SPHK1, SRC, STAT3*
GO:0031347	Regulation of defense response	15	*BTK, FYN, HSPD1, LEP, LYN, MAPK14, PDPK1, PRKCA, SNCA, SNX4, SPHK1, SRC, STAT3, TNFRSF11A, VAV1*
GO:0030335	Positive regulation of cell migration	15	*CXCL16, FER, HSPB1, ICAM1, IGFBP5, LYN, MAPK14, MSTN, PDPK1, POSTN, PRKCA, SELP, SPHK1, SRC, STAT3*
GO:0006979	Response to oxidative stress	14	*BTK, CASP3, FER, FYN, HSPB1, HSPD1, HTRA2, LPO, NME2, PDPK1, PPIF, SNCA, SPHK1, SRC*
GO:0030162	Regulation of proteolysis	14	*CNTN2, EFNA3, FGFR4, FYN, HSPD1, HTRA2, LYN, MAPK14, METAP1, PRKCA, SNCA, SRC, STAT3, UBE2G2*
GO:0040008	Regulation of growth	14	*CAMK2D, CXCL16, EIF4G2, HAMP, HTRA2, IGFBP3, IGFBP5, LEP, MAPK14, MSTN, SHC1, SPHK1, STAT3, TKT*
GO:0080135	Regulation of cellular response to stress	13	*CAMK2A, CAMK2D, DNAJB1, FYN, HSPB1, HTRA2, LEP, LYN, MAPKAPK2, NAMPT, SPHK1, TNFRSF11A, UBE2G2*
GO:0001817	Regulation of cytokine production	13	*BTK, CD84, HSPB1, HSPD1, LEP, LYN, MAPK14, MAPKAPK2, PDE4D, POSTN, SPHK1, SRC, STAT3*
GO:0043410	Positive regulation of MAPK cascade	12	*FGFR4, ICAM1, IGFBP3, LEP, MAPK14, MAPKAPK2, PDE5A, PRKCA, SHC1, SPHK1, SRC, TNFRSF11A*
GO:0010608	Posttranscriptional regulation of gene expression	12	*EIF4G2, EIF4H, EIF5A, HSPB1, IGFBP5, MAPK14, MAPKAPK2, METAP1, PRKCA, SMAD2, STAT3, YWHAZ*
GO:0044057	Regulation of system process	12	*CAMK2D, HAMP, ICAM1, IGFBP5, LEP, MSTN, PDE4D, PDE5A, PRKCA, REN, SPHK1, SRC*
WP:304	Kit receptor signaling pathway	11	*BTK, FYN, GRB2, LYN, MAPK14, PRKCA, PRKCB, SHC1, SRC, STAT3, VAV1*
GO:0034612	Response to tumor necrosis factor	11	*CASP3, COMMD7, CXCL16, HAMP, ICAM1, MAPK14, POSTN, SPHK1, TNFRSF11A, TNFRSF17, TNFRSF6B*
GO:0009895	Negative regulation of catabolic process	11	*EIF4G2, FYN, HTRA2, LEP, MAPK14, MAPKAPK2, METAP1, NAMPT, SNCA, STAT3, UBE2G2*
GO:0072593	Reactive oxygen species metabolic process	10	*FYN, GRB2, HSPD1, ICAM1, LEP, LPO, MAPK14, SNCA, STAT3, VAV1*
WP:2380	Brain-Derived Neurotrophic Factor (BDNF) signaling pathway	10	*CAMK2A, CASP3, CFL1, FYN, GRB2, MAPK14, PDPK1, SHC1, SRC, STAT3*
GO:0034248	Regulation of cellular amide metabolic process	10	*CASP3, EIF4G2, EIF4H, EIF5A, HSPB1, IGFBP5, METAP1, PDPK1, SPHK1, STAT3*
GO:0002703	Regulation of leukocyte mediated immunity	10	*BTK, CD84, FER, HSPD1, ICAM1, LEP, LYN, PDPK1, SNX4, VAV1*
WP:382	MAPK signaling pathway	9	*CASP3, FGF5, FGFR4, GRB2, HSPB1, MAPK14, MAPKAPK2, PRKCA, RAC3*
GO:0009743	Response to carbohydrate	9	*CASP3, ICAM1, LEP, LYN, NAMPT, NME2, PRKCA, PRKCB, SMAD2*
WP:289	Myometrial relaxation and contraction pathways	9	*CAMK2A, CAMK2D, IGFBP3, IGFBP5, IGFBP6, PDE4D, PRKCA, PRKCB, YWHAZ*
GO:0036293	Response to decreased oxygen levels	9	*CASP3, HSPD1, ICAM1, LEP, NAMPT, PDPK1, PKM, POSTN, SRC*
GO:0009408	Response to heat	9	*CAMK2A, CAMK2D, DNAJB1, HSPD1, HTRA2, LYN, MAPKAPK2, MSTN, PRKCA*
GO:0051896	Regulation of protein kinase B signaling	9	*FGF5, FGFR4, FYN, GRB2, IGFBP5, LEP, MSTN, SRC, VAV1*
GO:0000302	Response to reactive oxygen species	8	*BTK, CASP3, FER, FYN, HSPD1, PPIF, SPHK1, SRC*
WP:2324	AGE/RAGE pathway	8	*CASP3, MAPK14, PRKCA, PRKCB, SHC1, SMAD2, SRC, STAT3*
GO:0033500	Carbohydrate homeostasis	8	*FGFR4, ICAM1, IGFBP5, LEP, NME2, PDPK1, PRKCA, STAT3*
GO:0051092	Positive regulation of NF-kappaB transcription factor activity	8	*BTK, CAMK2A, FER, ICAM1, PRKCB, SPHK1, STAT3, TNFRSF11A*
WP:2203	Thymic Stromal LymphoPoietin (TSLP) signaling pathway	7	*BTK, FYN, LYN, MAPK14, SRC, STAT3, YES1*
WP:286	IL-3 signaling pathway	7	*FYN, GRB2, LYN, SHC1, SRC, STAT3, VAV1*
WP:2332	Interleukin-11 signaling pathway	7	*FYN, GRB2, ICAM1, PDPK1, SRC, STAT3, YES1*
GO:0014896	Muscle hypertrophy	7	*CAMK2D, HAMP, IGFBP5, LEP, MSTN, PDE5A, PRKCA*
GO:0010522	Regulation of calcium ion transport into cytosol	7	*CAMK2D, FYN, LYN, PDE4D, PDPK1, PRKCA, SNCA*
WP:481	Insulin signaling	6	*GRB2, MAPK14, PDPK1, PRKCA, PRKCB, SHC1*
WP:4298	Viral acute myocarditis	6	*CASP3, EIF4G2, FYN, RAC3, SRC, STAT3*
WP:4747	Netrin-UNC5B signaling pathway	6	*CASP3, FYN, ICAM1, MAPK14, PRKCA, SRC*
GO:0010675	Regulation of cellular carbohydrate metabolic process	6	*IGFBP3, LEP, PDPK1, SNCA, SRC, STAT3*
GO:0014743	Regulation of muscle hypertrophy	6	*CAMK2D, HAMP, IGFBP5, MSTN, PDE5A, PRKCA*
WP:400	p38 MAPK signaling pathway	5	*GRB2, HSPB1, MAPK14, MAPKAPK2, SHC1*
WP:2018	RANKL/RANK (receptor activator of NFKB (ligand)) signaling pathway	5	*ICAM1, LYN, MAPK14, SRC, TNFRSF11A*
GO:0014812	Muscle cell migration	5	*IGFBP3, IGFBP5, MSTN, POSTN, SRC*
WP:2032	Human Thyroid Stimulating Hormone (TSH) signaling pathway	5	*MAPK14, PDE4D, PDPK1, SRC, STAT3*
WP:2371	Parkinson’s disease pathway	5	*CASP3, HTRA2, MAPK14, SNCA, UBE2G2*
GO:0045123	Cellular extravasation	5	*FER, ICAM1, LEP, SELL, SELP*
GO:0051193	Regulation of cofactor metabolic process	4	*FYN, PDPK1, SNCA, STAT3*
GO:0060416	Response to growth hormone	4	*IGFBP5, LYN, NME2, STAT3*
GO:0070741	Response to interleukin-6	4	*FER, HAMP, ICAM1, STAT3*
WP:2038	Regulation of microtubule cytoskeleton	4	*MAPKAPK2, PRKCA, SRC, STAT3*
WP:3668	Hypothesized pathways in pathogenesis of cardiovascular disease	4	*MAPK14, POSTN, SHC1, SMAD2*
GO:0010543	Regulation of platelet activation	4	*LYN, PRKCA, SELP, TEC*
WP:4357	NRF2-ARE regulation	4	*FYN, PRKCA, SRC, YES1*
WP:1528	Physiological and pathological hypertrophy of the heart	4	*CAMK2D, MAPK14, PRKCB, STAT3*

## Supplementary Materials

The following are available online at https://www.mdpi.com/1422-0067/21/24/9540/s1.

## References

[1] Ohlendieck K. (2010). Proteomics of skeletal muscle differentiation, neuromuscular disorders and fiber aging. Expert Rev. Proteom..

[2] López-Otín C., Blasco M.A., Partridge L., Serrano M., Kroemer G. (2013). The hallmarks of aging. Cell.

[3] Bratic A., Larsson N.-G. (2013). The role of mitochondria in aging. J. Clin. Investig..

[4] Dai D.-F., Rabinovitch P.S., Ungvari Z. (2012). Mitochondria and cardiovascular aging. Circ. Res..

[5] Green D.R., Galluzzi L., Kroemer G. (2011). Mitochondria and the autophagy–inflammation–cell death axis in organismal aging. Science.

[6] Zane A.C., Reiter D.A., Shardell M., Cameron D., Simonsick E.M., Fishbein K.W., Studenski S.A., Spencer R.G., Ferrucci L. (2017). Muscle strength mediates the relationship between mitochondrial energetics and walking performance. Aging Cell.

[7] Choi S., Reiter D.A., Shardell M., Simonsick E.M., Studenski S., Spencer R.G., Fishbein K.W., Ferrucci L. (2016). 31P magnetic resonance spectroscopy assessment of muscle bioenergetics as a predictor of gait speed in the Baltimore Longitudinal Study of Aging. J. Gerontol. Ser. A Biomed. Sci. Med. Sci..

[8] Adelnia F., Urbanek J., Osawa Y., Shardell M., Brennan N.A., Fishbein K.W., Spencer R.G., Simonsick E.M., Schrack J.A., Ferrucci L. (2019). Moderate-to-Vigorous Physical Activity Is Associated With Higher Muscle Oxidative Capacity in Older Adults. J. Am. Geriatr. Soc..

[9] Zampino M., Brennan N.A., Kuo P.-L., Spencer R.G., Fishbein K.W., Simonsick E.M., Ferrucci L. (2020). Poor mitochondrial health and systemic inflammation? Test of a classic hypothesis in the Baltimore Longitudinal Study of Aging. GeroScience.

[10] Ubaida-Mohien C., Lyashkov A., Gonzalez-Freire M., Tharakan R., Shardell M., Moaddel R., Semba R.D., Chia C.W., Gorospe M., Sen R. (2019). Discovery proteomics in aging human skeletal muscle finds change in spliceosome, immunity, proteostasis and mitochondria. Elife.

[11] Adelnia F., Ubaida-Mohien C., Moaddel R., Shardell M., Lyashkov A., Fishbein K.W., Aon M.A., Spencer R.G., Ferrucci L. (2020). Proteomic signatures of in vivo muscle oxidative capacity in healthy adults. Aging Cell.

[12] Peruzzi F., Prisco M., Dews M., Salomoni P., Grassilli E., Romano G., Calabretta B., Baserga R. (1999). Multiple signaling pathways of the insulin-like growth factor 1 receptor in protection from apoptosis. Mol. Cell. Biol..

[13] Marzetti E., Calvani R., Bernabei R., Leeuwenburgh C. (2012). Apoptosis in skeletal myocytes: A potential target for interventions against sarcopenia and physical frailty–a mini-review. Gerontology.

[14] Friedman J.M. (2019). Leptin and the Endocrine Control of Energy Balance. Nat. Metab..

[15] Yamagishi S.-I., Edelstein D., Du X.-L., Kaneda Y., Guzmán M., Brownlee M. (2001). Leptin induces mitochondrial superoxide production and monocyte chemoattractant protein-1 expression in aortic endothelial cells by increasing fatty acid oxidation via protein kinase A. J. Biol. Chem..

[16] Ahima R.S., Flier J.S. (2000). Leptin. Annu. Rev. Physiol..

[17] Herzig S., Shaw R.J. (2018). AMPK: Guardian of metabolism and mitochondrial homeostasis. Nat. Rev. Mol. Cell Biol..

[18] Kreuder J., Borkhardt A., Repp R., Pekrun A., Göttsche B., Gottschalk U., Reichmann H., Schachenmayr W., Schlegel K., Lampert F. (1996). Inherited metabolic myopathy and hemolysis due to a mutation in aldolase A. N. Engl. J. Med..

[19] Merkulova M., Hurtado-Lorenzo A., Hosokawa H., Zhuang Z., Brown D., Ausiello D.A., Marshansky V. (2011). Aldolase directly interacts with ARNO and modulates cell morphology and acidic vesicle distribution. Am. J. Physiol. Cell Physiol..

[20] Yaku K., Okabe K., Nakagawa T. (2018). NAD metabolism: Implications in aging and longevity. Ageing Res. Rev..

[21] Lin S.-J., Guarente L. (2003). Nicotinamide adenine dinucleotide, a metabolic regulator of transcription, longevity and disease. Curr. Opin. Cell Biol..

[22] Huang Z., Ruan H.-B., Xian L., Chen W., Jiang S., Song A., Wang Q., Shi P., Gu X., Gao X. (2014). The stem cell factor/Kit signalling pathway regulates mitochondrial function and energy expenditure. Nat. Commun..

[23] Fernandez-Marcos P.J., Auwerx J. (2011). Regulation of PGC-1α, a nodal regulator of mitochondrial biogenesis. Am. J. Clin. Nutr..

[24] Leone T.C., Lehman J.J., Finck B.N., Schaeffer P.J., Wende A.R., Boudina S., Courtois M., Wozniak D.F., Sambandam N., Bernal-Mizrachi C. (2005). PGC-1α deficiency causes multi-system energy metabolic derangements: Muscle dysfunction, abnormal weight control and hepatic steatosis. PLoS Biol..

[25] Semba R.D., Nicklett E.J., Ferrucci L. (2010). Does accumulation of advanced glycation end products contribute to the aging phenotype?. J. Gerontol. Ser. A Biomed. Sci. Med. Sci..

[26] Thompson H., Scordilis S., Clarkson P., Lohrer W. (2001). A single bout of eccentric exercise increases HSP27 and HSC/HSP70 in human skeletal muscle. Acta Physiol. Scand..

[27] Davis T.L., Walker J.R., Campagna-Slater V., Finerty P.J., Paramanathan R., Bernstein G., MacKenzie F., Tempel W., Ouyang H., Lee W.H. (2010). Structural and biochemical characterization of the human cyclophilin family of peptidyl-prolyl isomerases. PLoS Biol..

[28] McArdle A., Vasilaki A., Jackson M. (2002). Exercise and skeletal muscle ageing: Cellular and molecular mechanisms. Ageing Res. Rev..

[29] McArdle A., Dillmann W.H., Mestril R., Faulkner J.A., Jackson M.J. (2004). Overexpression of HSP70 in mouse skeletal muscle protects against muscle damage and age-related muscle dysfunction. FASEB J..

[30] Doran P., Donoghue P., O’Connell K., Gannon J., Ohlendieck K. (2009). Proteomics of skeletal muscle aging. Proteomics.

[31] López-Armada M.J., Riveiro-Naveira R.R., Vaamonde-García C., Valcárcel-Ares M.N. (2013). Mitochondrial dysfunction and the inflammatory response. Mitochondrion.

[32] Sun N., Youle R.J., Finkel T. (2016). The mitochondrial basis of aging. Mol. Cell.

[33] Kim E.K., Choi E.-J. (2010). Pathological roles of MAPK signaling pathways in human diseases. Biochim. Biophys. Acta (BBA) Mol. Basis Dis..

[34] Basisty N., Kale A., Jeon O.H., Kuehnemann C., Payne T., Rao C., Holtz A., Shah S., Sharma V., Ferrucci L. (2020). A proteomic atlas of senescence-associated secretomes for aging biomarker development. PLoS Biol..

[35] Coppé J.-P., Desprez P.-Y., Krtolica A., Campisi J. (2010). The senescence-associated secretory phenotype: The dark side of tumor suppression. Annu. Rev. Pathol. Mech. Dis..

[36] Zampino M., Ferrucci L., Semba R.D. (2020). Biomarkers in the path from cellular senescence to frailty. Exp. Gerontol..

[37] Wiley C.D., Velarde M.C., Lecot P., Liu S., Sarnoski E.A., Freund A., Shirakawa K., Lim H.W., Davis S.S., Ramanathan A. (2016). Mitochondrial dysfunction induces senescence with a distinct secretory phenotype. Cell Metab..

[38] Fujita Y., Taniguchi Y., Shinkai S., Tanaka M., Ito M. (2016). Secreted growth differentiation factor 15 as a potential biomarker for mitochondrial dysfunctions in aging and age-related disorders. Geriatr. Gerontol. Int..

[39] Tanaka T., Biancotto A., Moaddel R., Moore A.Z., Gonzalez-Freire M., Aon M.A., Candia J., Zhang P., Cheung F., Fantoni G. (2018). Plasma proteomic signature of age in healthy humans. Aging Cell.

[40] Guralnik J.M., Fried L.P., Simonsick E.M., Lafferty M.E., Kasper J.D. (1995). The Women’s Health and Aging Study: Health and Social Characteristics of Older Women with Disability.

[41] Lohman T.G., Roche A.F., Martorell R. (1988). Anthropometric Standardization Reference Manual.

[42] Candia J., Cheung F., Kotliarov Y., Fantoni G., Sellers B., Griesman T., Huang J., Stuccio S., Zingone A., Ryan B.M. (2017). Assessment of variability in the SOMAscan assay. Sci. Rep..

[43] Kim C.H., Tworoger S.S., Stampfer M.J., Dillon S.T., Gu X., Sawyer S.J., Chan A.T., Libermann T.A., Eliassen A.H. (2018). Stability and reproducibility of proteomic profiles measured with an aptamer-based platform. Sci. Rep..

[44] Joshi A., Mayr M. (2018). In Aptamers They Trust: The Caveats of the SOMAscan Biomarker Discovery Platform from SomaLogic. Circulation.

[45] Cheung F., Fantoni G., Conner M., Sellers B.A., Kotliarov Y., Candia J., Stagliano K., Biancotto A. (2017). Web tool for navigating and plotting SomaLogic ADAT files. J. Open Res. Softw..

[46] Coen P.M., Jubrias S.A., Distefano G., Amati F., Mackey D.C., Glynn N.W., Manini T.M., Wohlgemuth S.E., Leeuwenburgh C., Cummings S.R. (2012). Skeletal muscle mitochondrial energetics are associated with maximal aerobic capacity and walking speed in older adults. J. Gerontol. Ser. A Biomed. Sci. Med. Sci..

[47] Paganini A., Foley J., Meyer R. (1997). Linear dependence of muscle phosphocreatine kinetics on oxidative capacity. Am. J. Physiol. Cell Physiol..

[48] Taylor D., Styles P., Matthews P., Arnold D., Gadian D., Bore P., Radda G. (1986). Energetics of human muscle: Exercise-induced ATP depletion. Magn. Reson. Med..

[49] Vanhamme L., Van Huffel S., Van Hecke P., van Ormondt D. (1999). Time-domain quantification of series of biomedical magnetic resonance spectroscopy signals. J. Magn. Reson..

[50] Naressi A., Couturier C., Castang I., De Beer R., Graveron-Demilly D. (2001). Java-based graphical user interface for MRUI, a software package for quantitation of in vivo/medical magnetic resonance spectroscopy signals. Comput. Biol. Med..

[51] Prompers J.J., Wessels B., Kemp G.J., Nicolay K. (2014). MITOCHONDRIA: Investigation of in vivo muscle mitochondrial function by 31P magnetic resonance spectroscopy. Int. J. Biochem. Cell Biol..

[52] Conley K.E., Jubrias S.A., Esselman P.C. (2000). Oxidative capacity and ageing in human muscle. J. Physiol..

[53] Arnold D., Matthews P., Radda G. (1984). Metabolic recovery after exercise and the assessment of mitochondrial function in vivo in human skeletal muscle by means of 31P NMR. Magn. Reson. Med..

[54] Meyerspeen M., Boesch C., Cameron D., Dezortova M., Forbes S.C., Heerschap A., Jeneson A.L.J., Kan H.E., Kent J., Layec G. (2020). P-31 magnetic resonance spectroscopy in skeletal muscle: Experts’ consensus recommendations. NMR Biomed..

[55] McCully K., Fielding R., Evans W., Leigh J., Posner J. (1993). Relationships between in vivo and in vitro measurements of metabolism in young and old human calf muscles. J. Appl. Physiol..

[56] Bindea G., Mlecnik B., Hackl H., Charoentong P., Tosolini M., Kirilovsky A., Fridman W.-H., Pagès F., Trajanoski Z., Galon J. (2009). ClueGO: A Cytoscape plug-in to decipher functionally grouped gene ontology and pathway annotation networks. Bioinformatics.

[57] Schafer M.J., Atkinson E.J., Vanderboom P.M., Kotajarvi B., White T.A., Moore M.M., Bruce C.J., Greason K.L., Suri R.M., Khosla S. (2016). Quantification of GDF11 and myostatin in human aging and cardiovascular disease. Cell Metab..

