# Supplementary material for: A Plasma Proteomic Signature of Skeletal Muscle Mitochondrial Function
Source: Int J Mol Sci. 2020 Dec 15;21(24):9540. doi: 10.3390/ijms21249540 (PMC7765442; doi:10.3390/ijms21249540)
Supplement: Supplementary file 1 [file ijms-21-09540-s001.zip › BLSA_GESTALT_SOMAlogic_appendix1.docx]

**Appendix 1. BLSA/GESTALT Exclusion and Inclusion criteria.**

BLSA/GESTALT Exclusion and Inclusion criteria:

- No established genetic diseases
- No self-reported difficulties or need for help in performing self-care or instrumental activities of daily living
- A score of 12 on the Short Physical Performance Battery of lower extremity function, or 10 or more in persons over 90 years of age
- Self-report of ability to walk 400 meters and no shortness of breath while performing normal activities of daily living, such as walking or climbing one flight of stairs
- No history of cardiovascular disease (including angina, myocardial infarction, congestive heart failure, cerebrovascular diseases; hypertension is allowed if controlled with treatment), diabetes, active cancer (except for locally limited non-melanoma skin cancer or successfully treated cancer without recurrence ten years before or longer), metabolic disease, severe hormonal dysfunction, neurological diseases, kidney or liver diseases, severe gastrointestinal diseases, musculoskeletal disorders (if they cause pathological weakness and/or chronic pain), sever psychiatric conditions, any medical condition that requires absolute need for long term treatment with antibiotics, antiviral, corticosteroids, immunosuppressant, H2 blockers, and pain medications, important sensory deficits and any condition that precludes participants from being tested with standard neuropsychological tests or providing informed consent
- No cognitive impairment based on standard neuropsychological testing (Blessed score <4, see <http://www.strokecenter.org/professionals/stroke-diagnosis/stroke-assessment-scales/> , or Mini Mental State Examination score >26 see <http://www.minimental.com/>)
- Labs:
  - White blood cells <12,000mcrL;
  - Platelets 100,000-600,000mcrL;
  - Hemoglobin >11g/dL;
  - Calculated creatinine clearance >50cc/min;
  - Bilirubin<1.5mg/dl unless higher levels can be ascribed to Gilbert’s diseas;
  - No abnormal level of SGOT and SGPT and alkaline phosphatase twice the normal serum concentrations
  - Albumin >3.4g/dL
